# Selective analysis of cell-free DNA in maternal blood for evaluation of fetal trisomy

**DOI:** 10.1002/pd.2922

**Published:** 2012-01-06

**Authors:** Andrew B Sparks, Eric T Wang, Craig A Struble, Wade Barrett, Renee Stokowski, Celeste McBride, Jacob Zahn, Kevin Lee, Naiping Shen, Jigna Doshi, Michel Sun, Jill Garrison, Jay Sandler, Desiree Hollemon, Patrick Pattee, Aoy Tomita-Mitchell, Michael Mitchell, John Stuelpnagel, Ken Song, Arnold Oliphant

**Affiliations:** 1 Aria Diagnostics, Inc.5945 Optical Court, San Jose, CA, 95138, USA; 2 Medical College of WisconsinMilwaukee, WI, 53226, USA

**Keywords:** DNA < fetal cells, nucleic acids and proteins, trisomy 21, trisomy 18, aneuploidy, next-generation sequencing, cell-free DNA

## Abstract

**Objective:**

To develop a novel prenatal assay based on selective analysis of cell-free DNA in maternal blood for evaluation of fetal Trisomy 21 (T21) and Trisomy 18 (T18).

**Methods:**

Two hundred ninety-eight pregnancies, including 39 T21 and seven T18 confirmed fetal aneuploidies, were analyzed using a novel, highly multiplexed assay, termed digital analysis of selected regions (DANSR™). Cell-free DNA from maternal blood samples was analyzed using DANSR assays for loci on chromosomes 21 and 18. Products from 96 separate patients were pooled and sequenced together. A standard *Z*-test of chromosomal proportions was used to distinguish aneuploid samples from average-risk pregnancy samples. DANSR aneuploidy discrimination was evaluated at various sequence depths.

**Results:**

At the lowest sequencing depth, corresponding to 204 000 sequencing counts per sample, average-risk cases where distinguished from T21 and T18 cases, with *Z* statistics for all cases exceeding 3.6. Increasing the sequencing depth to 410 000 counts per sample substantially improved separation of aneuploid and average-risk cases. A further increase to 620 000 counts per sample resulted in only marginal improvement. This depth of sequencing represents less than 5% of that required by massively parallel shotgun sequencing approaches.

**Conclusion:**

Digital analysis of selected regions enables highly accurate, cost efficient, and scalable noninvasive fetal aneuploidy assessment. © 2012 John Wiley & Sons, Ltd.

## INTRODUCTION

Prenatal testing for fetal chromosomal aneuploidies is commonly practiced and endorsed by professional medical organizations.^1^,^2^ Prenatal testing currently encompasses both screening and diagnostic modalities. Screening involves analysis of serum markers and/or ultrasound interpretation of fetal measurements such as nuchal translucency, but has suboptimal sensitivity and specificity.^1^ Diagnostic testing includes invasive procedures such as chorionic villus sampling (CVS) or amniocentesis, and although these tests are highly accurate, they come at significant health risks to the fetus and mother, including the potential loss of a healthy fetus.^3^

For the past several decades, numerous efforts have been pursued to develop a maternal blood test with improved accuracy for the detection of major fetal aneuploidies. Such blood tests could improve current screening practices for fetal aneuploidy. Initial efforts targeting isolation and analysis of circulating fetal cells in the maternal bloodstream have not proven successful, because of the challenges in detecting sufficient fetal cell numbers in circulation.^4^,^5^,^6^ By contrast, analysis of cell-free DNA (cfDNA) in maternal circulation has shown promise for evaluation of fetal aneuploidy. Several groups have demonstrated the use of massively parallel DNA shotgun sequencing (MPSS) to assay for fetal Trisomy 21 (T21) from cfDNA in maternal blood.^7^,^8^,^9^,^10^ More recently, several pilot studies have shown the ability of MPSS to assay for Trisomy 18 (T18) and Trisomy 13 (T13) from cfDNA in maternal blood, although with more variable results.^10^,^11^

Whereas MPSS has demonstrated robust technical performance, its cost and complexity, including the complexity of data analysis, creates challenges for broad clinical adoption. Because MPSS analyzes random genomic fragments from all chromosomes, large amounts of unutilized sequencing data are generated. For example, in one study involving pregnant women with T21 fetuses, a mean of 10 800 000 sequencing reads per sample were generated, but on average, only 32 000 (0.3%) chromosome 21 sequences were utilized for aneuploidy detection.^7^ Efforts to enrich for cfDNA from chromosomes of interest prior to MPSS have been described, but to date these approaches have not been used for detection of any genetic conditions.^12^

To address these limitations, we have developed a method called digital analysis of selected regions (DANSR™), which selectively evaluates specific genomic fragments from cfDNA. By enabling selective analysis of cfDNA, DANSR provides for more efficient use of sequencing to evaluate fetal aneuploidy. We report our initial investigation of this novel and promising method in a study of 298 pregnant women.

## METHODS

### Study population

A cohort comprised of women with singleton pregnancies was prospectively enrolled at selected prenatal care centers in the United States. Institutional Review Board approval was obtained for the study at all participating centers, and appropriate informed consent was obtained for all study participants. Average-risk women that had not undergone any invasive testing at the time of blood collection were enrolled and constituted the average-risk cases. The confirmed aneuploid cases consisted of pregnant women with T21 and T18 pregnancies confirmed via invasive testing with confirmatory fluorescent *in situ* hybridization and/or karyotype analysis.

### Sample preparation

An average of 8 mL of blood was collected from each subject into a Cell-free™ DNA tube (Streck) or ethylenediaminetetraacetic acid (EDTA) tube. An average of 5 mL of plasma was isolated from each sample via a double centrifugation protocol of 1600 ×*g* for 10 min, followed by 16 000 ×*g* for 10 min, after a tube transfer following the first spin. cfDNA was isolated from plasma using the Dynabeads® Viral NA DNA purification kit (Dynal) protocol, with minor modifications. An average of 17 ng cfDNA was isolated from each patient sample and arrayed into individual wells of a 96-well microtiter plate.

### Digital analysis of selected regions assay

**Figure 1:**
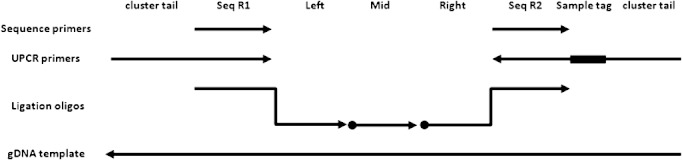
Schematic of DANSR assay. Arrows and dots indicate 3′OH and 5′PO_4_ moieties, respectively. When the left, middle, and right ligation oligos hybridize to their cognate genomic DNA (gDNA) sequences, their termini form two nicks. Ligation of these nicks results in the creation of an amplifiable template using the indicated UPCR primers. UPCR with 96 distinct right UPCR primers enables pooling and simultaneous sequencing of 96 different UPCR products on a single lane. The UPCR primers also contain left (TAATGATACGGCGACCACCGA) and right (ATCTCGTATGCCGTCTTCTGCTTGA) cluster tail sequences that support cluster amplification. Sequencing of the locus specific 56 bases and the seven sample specific bases are accomplished using read one (GATCTACACCGGCGTTATGCGTCGAGAC) and read two primers (TCAAGCAGAAGACGGCATACGAGAT) respectively

**Figure 2:**
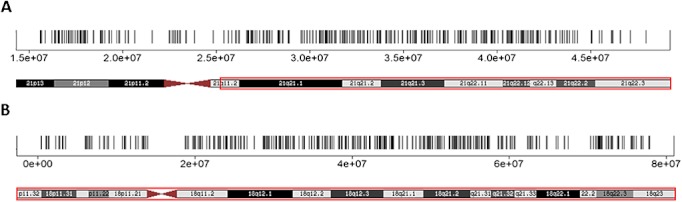
Chromosomal locations of selected loci. The selected loci are plotted as vertical lines against the Genome Reference Consortium human build 37 chromosome 21 in panel A and chromosome 18 in panel B. The plots’ physical spans are indicated with boxes on their respective karyograms

Digital analysis of selected regions enables simultaneous quantification of hundreds of loci by cfDNA-dependent catenation of two locus-specific oligonucleotides via an intervening ‘bridge’ oligo to form a PCR template. We designed DANSR assays corresponding to 384 loci on each of chromosomes 18 and 21 (Figure 1). We selected loci to have sequences unique to the chromosomes of interest, to have uniform locus-specific oligo melting temperatures, to have minimal complementarity with universal amplification sequences, and to not coincide with known polymorphisms and copy number variants. No explicit selection for chromosome location was employed; Figure 2 depicts the distribution of the selected loci along each chromosome.

Three oligonucleotides (IDT) per locus were used: a left oligo consisting of a 5′ universal amplification tail (TACACCGGCGTTATGCGTCGAGAC) followed by a locus-specific left sequence, a 5′-phosphorylated middle oligo, and a right oligo consisting of a 5′-phosphorylated locus-specific right sequence followed by a 3′ universal amplification tail (ATTGCGGGGACCGATGATCGCGTC). Oligonucleotides were pooled together to create a single DANSR assay oligo pool.

Cell-free DNA in each well of the microtiter plate was end labeled by incubation at 37 °C for 1 h in a 30 μL reaction containing 150 pmol biotin-16-dUTP (Roche), 12U TdT (Enzymatics) and 1X TdT buffer (Enzymatics). After isopropanol precipitation to remove free biotin, cfDNA was resuspended in 10 mM Tris-HCl pH8.0, 0.1 mM EDTA (TE) and immobilized onto 100 µg myOneC1™ magnetic beads (Dynal) in a 50 μL hybridization reaction consisting of 60 mM Tris-HCl pH8.0, 6 mM EDTA, 300 mM NaCl_2_, 35% formamide, 0.1% Tween-80, and 4 nM each locus specific DANSR oligonucleotides. The mixture was heated to 70 °C and annealed to 25 °C over 2 h, after which the beads were magnetically immobilized to the side of the microtiter plate well. After washing with 50 μL wash buffer (10 mM Tris-HCl pH 8.0, 1 mM EDTA, 50 mM NaCl_2_), the beads were resuspended in a 50 μL reaction containing 1 U Taq ligase (Enzymatics) and 1X Taq ligase buffer (Enzymatics) and incubated at 37 °C for 1 h. The beads were washed twice with wash buffer and resuspended in 30 μL TE. The ligation product was eluted from the beads by incubation at 95 °C for 3 min and amplified by PCR for 26 cycles in a 50 μL reaction containing 1U Pfusion polymerase (Fermentas), 1X Pfusion buffer, 200 μM dNTPs (Enzymatics), 200 nM left universal PCR (UPCR) primer (TAATGATACGGCGACCACCGAGATCTACACCGGCGTTATGCGTCGAGAC), and 200 nM right UPCR primer (TCAAGCAGAAGACGGCATACGAGATNNNNNNNAAACGACGCGATCATCGGTCCCCGCAAT), where NNNNNNN represents a 7 base sample index enabling 96 sample multiplexed sequencing. UPCR products from a single 96-well microtiter plate were pooled in equal volume, and the pool of UPCR products (library) was purified with AMPure XP (Beckman), according to the manufacturer's instructions. Each purified library was used as a template for cluster amplification on a TruSeq™ v2 SR cluster-kit flow-cell (Illumina), according to the manufacturer's protocols. The slide was processed on an Illumina HiSeq 2000™ to produce a 56 base locus-specific sequence and a 7 base sample tag sequence from each cluster.

### Data analysis

Sequencing reads with fewer than three mismatches against the expected sequence for each selected genomic location were counted. The median percentage of sequence reads that mapped to selected loci was 96%. Sequence counts within a sample were highly uniform across loci; on average, the sequence counts within a sample of 90% of loci fell within a 2.14-fold range. Sequence counts were normalized by systematically removing sample and genomic location biases via median polish.^13^,^14^ Specifically, the median count per locus within each sample was scaled to 1000. The counts were then log transformed to make chromosome, locus, and sample biases additive. The log transformed data were summarized using the linear model,


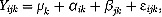


where *Y_ijk_* is the log count for locus *i*, sample *j* and chromosome *k*, *μ_k_* is the bias associated with chromosome *k*, *α_ik_* is the bias associated with location *i* on chromosome *k*, *β_jk_* is the bias associated with sample *j* at chromosome *k*, and *ε_ijk_* represents the error residuals in the model. The parameters of this model were estimated using the robust median polish technique.^13^

Once bias terms were estimated, normalization consisted of modifying terms to remove the associated biases. Systematic biases associated with chromosomes and loci were considered irrelevant for DANSR and were normalized by setting *μ_k_* to an arbitrary value (log_2_(1000)) and *α_ik_* = 0, respectively. The sample by chromosome interaction *β_jk_* is essential for capturing aneuploid status and was therefore retained in the model. The residuals *ε_ijk_* were also maintained. After modifying bias terms, the adjusted *Y_ijk_* values were antilogged to generate normalized counts for the downstream analysis.

Normalized sequence counts from assays for 384 genomic regions on chromosomes 18 and 21 were used to calculate a standard *Z*-test of proportions


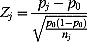


where *p_j_* is the observed proportion of representation for a given chromosome of interest in a given sample *j*, *p*_0_ is the median expected proportion for the given test chromosome, and *n_j_* is the sum of the mean count for each chromosome. *p_j_* was obtained by taking the 20% trimmed mean count across all chromosome *k* loci for sample *j* and dividing by the 20% trimmed mean count across all loci (both chromosomes 21 and 18) for sample *j*, and *p*_0_ was defined as the median of all *p_j_*'s within a sequencing lane. Because *p_j_* and *p*_0_ were computed after the normalization, no additional adjustments or corrections were made. *Z*-statistic standardization was performed via an iterative censoring approach. At each iteration, the samples outside of three median absolute deviations were removed. At the end of ten iterations, the mean and standard deviation were calculated using only the uncensored samples. All samples were then standardized against this mean and standard deviation. The Kolmogorov–Smirnov test,^15^ and Shapiro–Wilk's test^16^ were used to test for the normality of the average-risk samples’ *Z* statistics.

The separation distance was calculated by taking the difference (in *Z* statistic) between the lower 5th percentile of the affected and the upper 95th percentile of the average-risk samples.

## RESULTS

A total of 298 plasma samples were analyzed in this study, including 39 T21 samples, seven T18 samples, and 252 samples from average-risk pregnant women. Table 1 summarizes demographic characteristics of the subjects included in the study. The median maternal age was 31 years (range 18–44 years) for all subjects. The median maternal ages for average-risk, T21, and T18 pregnancy subjects were 30 years (range 18–41), 36 years (range 18–43), and 36 years (range 29–44), respectively. The overall median gestational age was 13.4 weeks. The median gestational ages for the average-risk, T21, and T18 pregnancy subjects were 12.9 weeks, 20.0 weeks, and 17.3 weeks, respectively. The higher median maternal and gestational ages for subjects with confirmed aneuploidy were expected, given that a portion of these women had already undergone invasive testing and represented a higher-risk population.

**Table 1:** Demographic characteristics of the 298 pregnant women

Clinical parameter	Classification	Total	Median	Mean	Range
Maternal age (years)	All	298	31	30.4	[18,44]
	Avg risk	252	30	29.6	[18,41]
	T21	39	36	34.2	[18,43]
	T18	7	36	37.3	[29,44]
Gestational age (weeks)	All	298	13.4	15.6	[7,35.4]
	Avg risk	252	12.9	14.8	[7,33.1]
	T21	39	20.0	20.5	[13.4,35.4]
	T18	7	17.3	17.9	[11.1,23.6]

**Figure 3:**
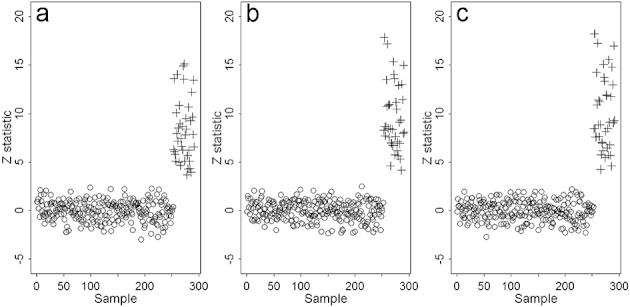
T21 *Z* statistics at various median per-sample counts. At each median per-sample count, the average-risk samples (open circles) were normally distributed around zero, while the T21 samples (crosses) were at least three standard deviations away. The separation distance was calculated by taking the difference (in *Z* statistic) between the 5th percentile of the affected samples and the 95th percentile of the average-risk samples. When the median per-sample count was 204 000 (a), the separation distance between average-risk and T21 samples was 4.2. When the median per-sample count was increased to 410 000 (b) and 620 000 (c), the resulting separation distances increased to 5.2 and 5.4, respectively

**Figure 4:**
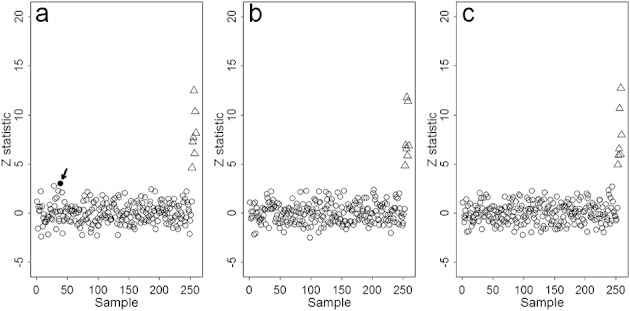
T18 *Z* statistics at various median per-sample counts. At a median per-sample count of 204 000 (a), the separation distance between average-risk samples (open circles) and T18 samples (triangles) was 4.9. When the median per-sample count was increased to 410 000 (b) and 620 000(c), the separation distances were both 5.2. When the median per-sample count was 204 000, one average-risk sample received a *Z* statistic of 3.005 (a; black dot, arrow). This sample subsequently received a *Z* statistic of less than three when we increased the median per-sample count to 410 000

Figure 3 shows the distribution of *Z* statistics for average-risk and T21 plasma samples at different sequencing depths. Figure 4 shows the distribution of *Z* statistics for average-risk and T18 plasma samples at different sequencing depths. In both sets of samples, the average-risk samples have a mean *Z* statistic close to zero, whereas the aneuploid samples have *Z* statistics greater than 3.6. The *Z* statistics for the average-risk samples is normally distributed; Kolmogorov and Shapiro–Wilk's tests of normality showed *p* < 0.50 and *p* < 0.13, respectively. The standard deviation of the *Z* statistic for average-risk samples was approximately one (range 0.99–1.02), as shown in Table 2. Figures 3 and 4 demonstrate that the separation between the average-risk and aneuploid samples increases as the median sequencing counts per sample increases. Using a *Z* statistic cut-off of three to distinguish between average-risk and aneuploid samples, all aneuploid samples were correctly classified. At the lowest sequencing depth, one of 252 average-risk samples (0.4%) had a *Z* statistic of 3.005 when evaluating for T18 (Figure 2(a)). At higher sequencing depths, this sample had a *Z* statistic < 3.

**Table 2:** Summary statistics of the samples at different sequencing depths. The median per-sample count is the median of total counts across all samples. The percentage of sequences mapped was calculated by dividing the number of sequences mapped by the total number of observed sequences. The separation distance was calculated by taking the difference (in *Z* statistic) between lower 5th percentile of the affected samples and the upper 95th percentile of the average-risk samples

Median per-sample count (×1000)	Median % mapped	T21 separation (SD)	T18 separation (SD)	Avg risk *Z* statistics SD
204	96.6	4.2	4.9	1.02
410	96.8	5.2	5.2	0.99
620	96.8	5.4	5.2	1.00

To quantify the distinction between euploid and aneuploid samples, we calculated the separation distance as the difference between the *Z* statistic value of the upper 95th percentile of the average-risk samples and the lower 5th percentile of the affected samples. The separation distance for T21 and T18 samples increases with sequencing depth (Table 2). The greatest benefit in separation distance for both T21 and T18 was realized when increasing the median sequencing counts per sample from 204 000 to 410 000. Increasing the sequencing counts per sample from 410 000 to 620 000 had marginal benefit for T21 and no statistical benefit for T18. The improved separation resulting from increasing sequence depth beyond 204 000 counts per sample is consistent with the finding that at the lowest depth of sequencing, one average-risk sample had a *Z* statistic slightly greater than three (3.005), whereas with deeper sequencing, the *Z* statistic of all average-risk samples was less than three.

## DISCUSSION

We have demonstrated that DANSR enables efficient and selective evaluation of cfDNA from maternal blood for fetal aneuploidy. We analyzed 298 plasma samples from pregnant women, including 39 T21 and seven T18 cases. Previous studies with MPSS have used a *Z* statistic cut-off of three standard deviations to classify cases as aneuploid or euploid.^7^,^9^ Using a similar statistical analysis, we correctly distinguished all aneuploid cases from average-risk cases using as few as 420 000 reads per sample.

Digital analysis of selected regions has several advantages compared with MPSS as an assay for aneuploidy. First, the fraction of raw sequencing reads that map to expected loci exceeds 96% with DANSR; by contrast, studies using MPSS report mapping rates of 20% to 50%.^7^,^8^ Second, DANSR produces sequence data only from chromosomes of clinical interest; by contrast, MPSS produces data from all chromosomes, irrespective of their relevance to analysis of aneuploidy. Taken together, the DANSR advantages of mapping efficiency and selective analysis of specific chromosomes result in a greater than tenfold improvement on the sequencing cost and throughput of MPSS. DANSR thus has the potential to significantly lower the cost of noninvasive prenatal testing, which may make it economically practical for broad clinical use.

The sequencing cost and throughput advantages conferred by DANSR do not come at the cost of additional process complexity; DANSR library preparation requires a similar amount of time and effort as MPSS library preparation. The DANSR assay entails five major steps: biotinylation, precipitation, hybridization, ligation, and PCR. By comparison, Illumina's library preparation protocol entails six steps: end repair, AMPure XP, adenylation, adaptor ligation, AMPure XP, and PCR.

Because MPSS uses DNA from the entire genome, it theoretically requires less blood/plasma/cfDNA per patient than DANSR. In this study, we analyzed samples with a range of 5 to 11 mL of blood, yielding a range of 2.8 to 6.5 mL plasma, and a range of 5 to 60 ng cfDNA. We observed no difference in performance between samples with small versus large quantities of blood, plasma, or cfDNA. Although this suggests a lower amount of input material could be used, a 10 mL blood draw is clinically reasonable and standard. One potential advantage of MPSS over selective analysis is that MPSS data is collected from all chromosomes, allowing for the theoretical possibility of identification of aneuploidy events involving any chromosome. In this study, we focused our analysis on chromosomes 18 and 21, and found that 384 loci per chromosome was sufficient to enable aneuploidy discrimination. We recently evaluated cfDNAs using a single DANSR pool consisting of assays for more than 5000 loci (data not shown), and observed no substantial adverse effects of higher assay multiplex on data quality. This suggests that DANSR could be extended to include other chromosomes of clinical relevance.

In this study, we examined the effect of depth of sequencing on the ability of DANSR to discriminate between average-risk and aneuploid cases. We demonstrated that increasing the depth of sequencing results in better separation between average-risk and aneuploid cases. The benefit of increased sequencing depth likely reflects sampling noise reduction at the individual locus level. The incremental benefit of increasing sequencing coverage needs to be weighed against the additional sequencing costs. However, since these data were generated, several sequencing technology companies have made recent strides toward higher throughput. For example, a single sequencing lane on the Illumina HiSeq 2000 using version three chemistry can produce 150 million high quality counts, which readily accommodates 96 samples even at the highest sequencing depth explored in this study.

Although this study demonstrates the promise of selective cfDNA analysis, there are several caveats. First, the putative nontrisomy cases in this study were comprised of average-risk women whose fetal ploidy status was not independently confirmed. However, because the average age of the normal cases was 30 years, the likelihood of a T21 or T18 fetus being present in this cohort was very low. Second, the confirmed aneuploid cases were of older gestational age. It has been reported that the amount of fetal DNA in maternal blood increases with gestational age, although the effect is most pronounced in the third trimester.^17^ Higher fetal DNA amounts may bias the results for the confirmed aneuploid cases, but the likelihood of this is low, because the majority of cases were in the second trimester or earlier. Future studies in which all the cases are confirmed (either by genotype or phenotype) to be diploid or triploid, and in which cases are matched for gestational age, are warranted. Lastly, this study was intentionally not blinded, because it represented our initial exploration of selective analysis of cfDNA for fetal aneuploidy using the DANSR assay. We are currently conducting prospective blinded studies to further validate the DANSR approach.

## CONCLUSION

Digital analysis of selected regions represents a novel and promising approach to prenatal testing. By enabling selective analysis of cfDNA for evaluation of fetal aneuploidy, DANSR has the potential to significantly reduce test costs compared with MPSS approaches. In addition, DANSR may enable the study of other genetic conditions, including subchromosomal copy number variations, which are not as amenable to analysis by techniques such as MPSS. Prioritization of which genetic conditions to evaluate will require consideration of medical necessity, and the practicality of obtaining sufficient clinical samples for validation.

**WHAT'S ALREADY KNOWN ABOUT THIS TOPIC?**

- Non-invasive detection of fetal trisomy is feasible using massively parallel shotgun sequencing, which evaluates cfDNA fragments without regard to their chromosome of origin.

**WHAT DOES THIS STUDY ADD?**

- This study demonstrates for the first time non-invasive detection of trisomy 21 and trisomy 18 using selective sequencing of cfDNA from specific chromosomes, a dramatically more efficient and scalable method.
